# Quasi-classical modeling of molecular quantum-dot cellular automata multidriver gates

**DOI:** 10.1186/1556-276X-7-274

**Published:** 2012-05-30

**Authors:** Ehsan Rahimi, Shahram Mohammad Nejad

**Affiliations:** 1 Nanoptronics Research Center, School of Electrical and Electronic Engineering, Iran University of Science and Technology, Tehran, 16844, Iran

**Keywords:** Electron transfer reactions, Molecular electronics, Molecular gates, Molecular quantum-dots, Quantum cellular automata

## Abstract

Molecular quantum-dot cellular automata (mQCA) has received considerable attention in nanoscience. Unlike the current-based molecular switches, where the digital data is represented by the on/off states of the switches, in mQCA devices, binary information is encoded in charge configuration within molecular redox centers. The mQCA paradigm allows high device density and ultra-low power consumption. Digital mQCA gates are the building blocks of circuits in this paradigm. Design and analysis of these gates require quantum chemical calculations, which are demanding in computer time and memory. Therefore, developing simple models to probe mQCA gates is of paramount importance. We derive a semi-classical model to study the steady-state output polarization of mQCA multidriver gates, directly from the two-state approximation in electron transfer theory. The accuracy and validity of this model are analyzed using full quantum chemistry calculations. A complete set of logic gates, including inverters and minority voters, are implemented to provide an appropriate test bench in the two-dot mQCA regime. We also briefly discuss how the QCADesigner tool could find its application in simulation of mQCA devices.

## Background

**Figure 1:**
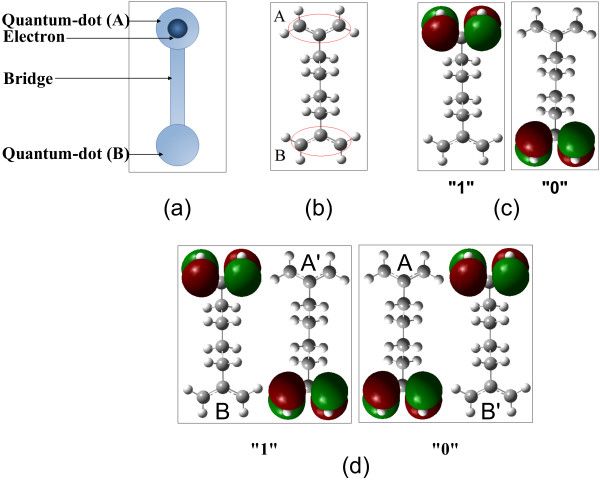
**Binary representation in the mQCAparadigm.** (**a**) Schematic structure of a two-dot mQCA cell. (**b**) A two-dot molecule. (**c**) In two-dot cells, depending on which of the upper (**A**) or lower (**B**) quantum-dot is occupied, binary “1” or “0” is represented. (**d**) In four-dot cells, binary “1” and “0” is represented within the occupation of AB’ or A’B dots correspondingly.

Recent advances in molecular electronics on one hand and the limitations of conventional semiconductor devices, on the other hand, have driven a surge of activities towards the realization of molecular devices, circuits, and systems. Achieving the ultimate diminution in size, power consumption, and delay of electronic devices and systems has always been a challenging endeavor of scientists and designers in this field. Due to the prospect of size reduction in electronics offered by molecular-level control of properties, molecular electronics provides means to extend the Moore's law beyond the foreseen limits of small-scale conventional silicon integrated circuits. The small size of molecules allows high device density in the range of 10^11^ to 10^12^ devices/cm^2^[1]. Besides, the chemical self-assembly capacity in manufacturing molecular devices provides many advantages to conventional semiconductor manufacturing technology, including lower manufacturing cost and uniform device reproducibility. Molecular electronics endeavors to use the nonlinear current–voltage characteristics of individual molecules or molecular assemblies as active devices (diodes, transistors, etc.) in electronic circuits. However, the power consumption of molecular current switches at very high frequencies is still a drawback [2]. The π-σ-π mixed-valence type molecules, which provide double-well potentials for electrons, have been proposed and studied by Aviram towards the synthesis of memory, logic, and amplification [3]. Lent proposed using molecules in representing binary information within the molecular quantum-dot cellular automata (mQCA) paradigm [1,4]. Molecular QCA provides an alternative approach to represent and process data, where binary representation lies in the charge configuration within molecules rather than in the on/off states of current switches. A cell in the mQCA model consists of a number of molecular quantum dots (or redox centers) and a few electrons. The electrons tend to occupy antipodal sites as a result of Coulomb repulsion. The Columbic interactions cause electrons to tunnel from one redox center to another in a cell, but not between cells. Thus, it is likely that no current flows, since the neighboring cells are coupled by electrostatic field. Figure 1 depicts two-dot and four-dot mQCA cells and how binary “1” or “0” is represented. The first QCA device was implemented and tested using metal dots at near 0 K [5]. Semiconductor implementation of QCA using GaAs/AlGaAs heterostructure materials has been reported in [6,7] as well. Molecules are good containers for keeping electric charge and mQCA cells have a more promising future to work at room temperature [8]. Nonbonding *π* or *d* orbitals of a single molecule (or multiple molecules) can function as quantum dots, where the electric charge is localized in each cell. Synthesis of two-dot and four-dot mixed-valence candidate molecules for mQCA has been reported in [9-14]. Many of these molecules are mixed-valence type and include transition metals to enable fast electron transfer reactions [15]. Molecular QCA gates are the building blocks of circuits in this paradigm. Calculation of the electronic structure of mQCA gates composed of these molecules is challenging, since the number of basis set functions grows exponentially as the number of molecules and atoms are increased. Besides, many of the *ab initio* methods fail in describing charge distribution in mixed-valence complexes. Therefore, developing semi-classical models to study mQCA gates is of high importance. Currently QCADesigner [16] utilizes nonlinear and two-state approximations to solve metallic-based QCA circuits. Several QCA circuits including combinational as well as sequential circuits have been studied using QCADesigner. Examples are adders, shift registers, RAM, digital data storage, and simple microprocessors [17-26]. In this paper, we center on two-dot mQCA and analyze the validity and accuracy of the two-state model approximation for studying multidriver mQCA gates. This study provides an approach to enhance the QCADesigner tool for simulation of mQCA devices in the future.

## Methods

### **Two-dot molecular QCA test bench**

**Figure 2:**
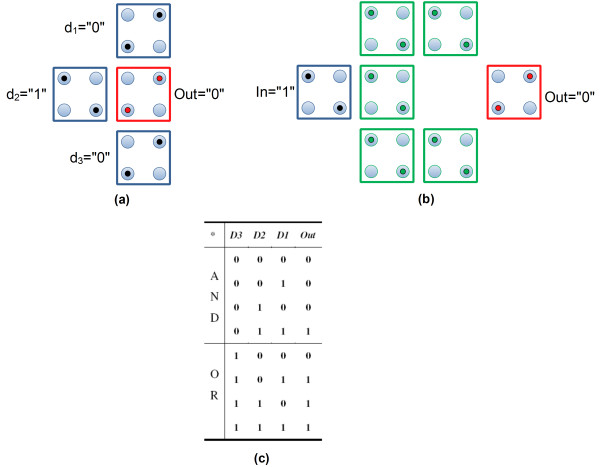
**Four-dot QCA gates.** (**a**) The universal MV gate. The majority of the three fixed inputs, which is “0” in this figure, appears at the output as a result of Coulombic interactions and minimum energetics. (**b**) The inverter gate. (**c**) Truth table of the MV gate. When d_3_ = ”0”, the MV gate performs AND logical operations on d_1_ and d_2,_ and when d_3_ = ”1” the MV gate functions as a two-input OR gate [25].

The majority voter (MV) and the inverter (INV) gates [25] are the fundamental building blocks of any circuit in the four-dot QCA architecture. These gates have been schematically shown in Figure 2a, b. Particularly, the MV gate is referred to as the universal QCA gate, since the AND and OR logical operations can be done by this gate, as evident from the truth table shown in Figure 2c.

**Figure 3:**
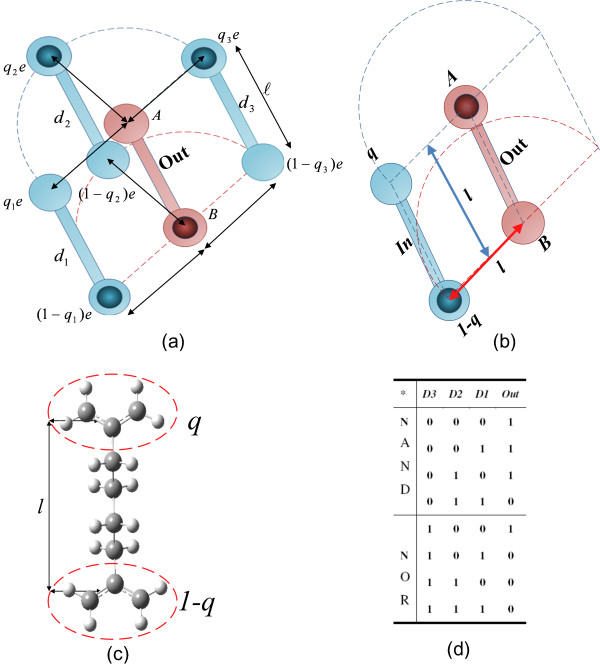
**Two-dot QCA gates.** (**a**) Structure of the three-input MinV gate. This gate is composed of three fixed inputs (d_1_, d_2_, and d_3_) and a two-dot molecule (AB) as output. The minority of the three fixed inputs, which is “0” in this figure, appears at the output. (**b**) When there is only one input, the MinV gate functions as an inverter gate. (**c**) Two point charges *q* and *1-q* separated by distance *l*, which is the distance between the middle of upper and lower π-bonds, are used to mimic a fixed input. (**d**) Truth table of the MinV gate. When d_3_ = ”0”, the MinV gate performs NAND logical operation on d_1_ and d_2,_ and when d_3_ = ”1” the MinV gate functions as a two-input NOR gate.

Our multidriver minority voter (MinV*)* gate is composed of *m* drivers, where *m* is an odd number, as inputs and one output. Figure 3a schematically illustrates the three-driver MinV model gate in the two-dot mQCA regime. When only one driver (e.g., the *d*_*1*_) is present, the model gate serves as an INV gate (Figure 3b). In the multidriver MinV model gate, all the distances between the centers of the molecules are *l*, which is equal to the distance between the middle of upper and lower *π*-bonds as shown in Figure 3c. The inputs of the gates are kept fixed, while the output cells switch to their stable states. To this end, two point charges *q* and *1-q* separated by distance *l* are used to mimic each input as depicted in Figure 3c.

The MinV gate is an alternative to the MV gate in the four-dot QCA, where the output is inverted. Compared to MV and INV gates in the four-dot architecture, which require 16 and 28 quantum-dots correspondingly, the MinV and INV gates require only 8 and 4 quantum-dots in the two-dot mQCA regime. Consequently, these gates provide a small two-dot mQCA test bench, which make high level quantum chemical calculations feasible. The MinV gate can perform NAND and NOR logical operations, as shown in Figure 3d, and provides a functionally complete logic set to implement any logic function in the two-dot mQCA framework. Additionally, it is possible to implement multi-input (or multidriver) MinV gates, which in turn decrease the total number of gates required to implement a logic circuit. It is important to note that since the MinV gate is not a planar gate, circuits implemented in the two-dot mQCA regime are not planar circuits. We highlight that the practical QCA circuits require clocked-control cells and clocking schemes [21,27-29], which are not addressed in this paper.

### **Two-state model for molecular QCA gates**

The charge configuration in a QCA cell is quantified by the so called ‘polarization’, and is defined as [30]

(1) $$P=\frac{\left(q_{A}+q_{B^{\prime }})-(q_{B}+q_{A^{\prime }}\right)}{q_{A}+q_{B}+q_{A^{\prime }}+q_{B^{\prime }}}$$

where *q*_*A*_*q*_*B*_*q*_*A′*_ and *q*_*B′*_ are the charges localized at four quantum dots labeled in Figure 1. For two-dot mQCA cells, the polarization is given by the charges *q*_*A*_ and *q*_*B*_ at the corresponding redox centers in Equation 1. The polarization of a QCA cell varies between −1 and 1, while negative and positive polarizations represent binary “0” and “1”, respectively. In two-dot mQCA cells, the normalized dipole moment of the used two-dot molecule is also identical to the polarization, which is given by

(2) $$P=\frac{\mu_{\alpha }}{l/2}$$

where μ_α_ denotes the component of the molecular dipole moment that is parallel to the bridge direction, and the origin is in the middle of the bridge. The dipole moment of an mQCA cell can be obtained through full quantum chemical calculations. An important parameter of a QCA device is the Kink energy (*E*_*k*_), which is the required energy to excite the system from the ground state to the first excited state. To distinguish a bit value from the thermal environment, *E*_*k*_ must be greater than *k*_*B*_*T*[31], where *T* is the operation temperature in Kelvin, and *k*_*B*_ is the Boltzmann's constant. The *E*_*k*_ represents the energy cost of cells *i* and *j* having opposite polarizations. That is, the electrostatic interaction between all the charges in cells *i* and *j* is calculated by [16]

(3) $$E_{i,j}=\sum_{i,j}\frac{1}{4\pi \epsilon_{0}\epsilon_{r}}\frac{q_{i}q_{j}}{\left|r_{i}-r_{j}\right|}$$

where *ϵ*_0_ is the permittivity of free space, and *ϵ*_*r*_ is the relative permittivity of the material system. The Kink energy is then given by [16]

(4) $$E_{k}={E^{\prime }}_{i,j}-E_{i,j}$$

where *E′*_*i,j*_ and *E*_*i,j*_ denote the electrostatic energy of cells *i* and *j* having opposite and same polarizations correspondingly.

Tougaw and Lent have used a simple Hamiltonian of the extended-Hubbard type to describe the dynamic behavior of four-dot metallic-based QCA nanodevices [32]. Although this Hamiltonian describes the dynamics of the coherent system composed of arrays of four-dot QCA cells elegantly in theory, it is only possible to model the small systems employing this scheme, since the total required number of direct-product basis sets grows exponentially with the number of cells. In other words, an array with *N* number of four-dot cells and *B* number of basis sets in each cell requires the total number of direct-product basis sets as [32]

(5) $$n_{\mathrm{basis}}=B^{N}$$

By reducing the number of basis sets for each cell and picking up the two orthogonal ones, the Hamiltonian of a four-dot QCA wire can be mapped to Ising model as [32,33]

(6) $$H=-\gamma \sum_{i=1}^{N}\sigma_{x}(i)-\frac{E_{k}}{2}\sum_{i=1}^{N-1}\sigma_{z}(i)\sigma_{z}(i+1)$$

where *E*_*k*_ is the kink energy of four-dot cells, *γ* is the tunneling energy, and σ_x_ and σ_z_ are Pauli spin matrices. In semiconductor and metal-dot QCA, the tunneling barriers of the cells are connected to electrodes, and their heights are controlled externally by voltage sources [33]. The steady-state polarization of any cell, *j* in a block of cells, can be obtained as a solution to the Hartree-Fock intercellular approximation. This approximation decouples the line of *N* cells into *N* single cell subsystems and assumes that the cells are only coupled through expectation values of polarizations. The consequent solution is [33],

(7) $$P_{j}=\frac{\frac{E_{k}}{2\gamma }{\bar{P}}_{j}}{\sqrt{1+{\left(\frac{E_{k}}{2\gamma }{\bar{P}}_{j}\right)}^{2}}}$$

where *P*^—^_*j*_ is the sum of the polarizations of the neighboring four-dot QCA cells. Equation 7 is currently used in the nonlinear and two-state simulation engine of QCADesigner to solve the metallic-based QCA circuits. It is important to note that mQCA utilizes non-abrupt clocking to reduce the probability of Kink, the property that is not currently present in the QCADesigner as it is based on metallic QCA. In mQCA, the tunneling barriers can be controlled by external electric field [27]. It is demanding to enhance the tool to be able to simulate mQCA circuits. As a primary step towards this end, we present how a similar equation to (7) can be derived directly from the two-state approximation in electron transfer theory [34,35] for two-dot mQCA. We then discuss how these approximations affect the results compared to those obtained from full quantum chemistry calculations.

Equation 8 describes the electron transfer (ET) process in a two-dot mQCA cell, where the two redox centers, *A* and *B*, are linked through an intervening bridge, *I*.

(8) $$A^{-}-I-B\Leftrightarrow A-I-B^{-}$$

The electronic coupling between the redox centers, which is time independent, is an important factor in the ET process. Within the two-state approximation, the Landau-Zener model [36,37] for avoided crossing of energy surfaces may be applied, where the two diabatic states “1” and “0” denoted by *ψ*_*a*_ and *ψ*_*b*_, and with energies *H*_*aa*_ and *H*_*bb*_ interact. The ground state *ψ*_*1*_ and the first excited state of a QCA cell, *ψ*_*2*_, can be related to the diabatic states *ψ*_*a*_ and *ψ*_*b*_ by a unitary transformation [34]

(9) $$\psi_{1}=\cos\eta \;\psi_{a}-\sin\eta \;\psi_{b}$$

(10) $$\psi_{2}=\sin\eta \;\psi_{a}+\cos\eta \;\psi_{b}$$

In Equations 9 and 10*ψ*_*1*_ and *ψ*_*2*_ are orthonormal, whereas *ψ*_*a*_ and *ψ*_*b*_ are not orthogonal in general. The correspondence between diabatic and adiabatic two-state models arises from the secular determinant (*S*_*ab*_*= <ψ*_*a*_*|ψ*_*b*_*>* is neglected) [38]

(11) $$\left|\begin{array}{cc} H_{\mathrm{aa}}-E & H_{\mathrm{ab}}\\ H_{\mathrm{ab}} & H_{\mathrm{bb}}-E \end{array}\right|=0$$

where *E* is the adiabatic energy eigenvalue. The *η* in Equations 9 and 10 satisfies [38]

(12) $$\tan2\eta =2H_{\mathrm{ab}}/\left(H_{\mathrm{aa}}-H_{\mathrm{bb}}\right)$$

The energy difference between the two diabatic states in the output cell of the MinV gate can be approximated by calculating the difference between the electrostatic energies of the gate for the two output configurations, where the unit charge is localized at sites *A* and *B* correspondingly. Using Equation 3, for the “1” and “0” output states (Figure 3a), we obtain

(13) $$H_{\mathrm{aa}}-H_{\mathrm{bb}}=\frac{1}{4\pi \epsilon_{0}}\left[\frac{(q_{1}+q_{2}+q_{3})e^{2}}{l}+\frac{(3-(q_{1}+q_{2}+q_{3}))e^{2}}{\sqrt{2}l}\right]-\frac{1}{4\pi \epsilon_{0}}\left[\frac{(q_{1}+q_{2}+q_{3})e^{2}}{\sqrt{2}l}+\frac{(3-(q_{1}+q_{2}+q_{3}))e^{2}}{l}\right]$$

Inserting Equation 13 into Equation 12 we have

(14) $$\cot2\eta =\left(\frac{1}{2H_{\mathrm{ab}}}\right)\left(\frac{e^{2}}{4\pi \epsilon_{0}l}\right)\left(\frac{2-\sqrt{2}}{2}\right)\left[2(q_{1}+q_{2}+q_{3})-3\right]$$

The Kink energy of two-dot cells can be calculated from Equation 3 and 4 for two neighboring cells as

(15) $$E_{k}=\frac{-e^{2}}{4\pi \epsilon_{0}l}\left(\frac{2-\sqrt{2}}{2}\right)$$

and for each driver, the polarization is defined using Equation 1

(16) $$P_{d_{i}}=\frac{q_{i}e-(1-q_{i})e}{q_{i}e+(1-q_{i})e}=2q_{i}-1\;\text{,}\;i=1\text{,}\;2\text{,}\;3$$

Thus, Equation 14 can be rewritten in terms of the Kink energy and the input polarizations

(17) $$\cot2\eta =\frac{-E_{k}}{2H_{\mathrm{ab}}}\sum_{i=1}^{3}P_{d_{i}}$$

And finally, using Equation 1 and the transformation coefficients in Equations 9 and 10, the output polarization of the MinV gate is obtained

(18) $$P_{o}=\frac{(+e)\cos^{2}\eta -(+e)\sin^{2}\eta }{(+e)\cos^{2}\eta +(+e)\sin^{2}\eta }=\cos2\eta =\frac{-\cot2\eta }{\sqrt{1+\cot^{2}2\eta }}$$

Inserting Equation 17 into Equation 18, we can find the polarization of the output cell of the MinV gate in terms of the polarizations of the inputs straightforwardly as

(19) $$P_{o}(P_{d_{1}},P_{d_{2}},P_{d_{3}})=\frac{\frac{E_{k}}{2H_{\mathrm{ab}}}\sum_{i=1}^{3}P_{d_{i}}}{\sqrt{1+{\left(\frac{E_{K}}{2H_{\mathrm{ab}}}\right)}^{2}{\left(\sum_{i=1}^{3}P_{d_{i}}\right)}^{2}}}$$

Equation 19 in two-dot mQCA is analogous to Equation 7 in four-dot metal-based QCA*,* where the tunneling energy *γ* appears as electronic coupling of redox centers (*H*_*ab*_) in Equation 19. They also imply

(20) $$P_{o}(d_{1},d_{2},d_{3})=P_{o}(d_{1}+d_{2}+d_{3})$$

The additivity relation in Equation 20 originates from the additivity of electrostatic potential energy in Equation 13 for diabatic states.

Multidriver MinV gates help reduce the number of needed gates for implementation of a logic circuit. Similarly, for an *m*-input MinV gate, we obtain

(21) $$P_{o}(P_{d_{1}},P_{d_{2}},\ldots ,P_{d_{m}})=P_{o}\left(\sum_{i=1}^{m}P_{d_{i}}\right)=\frac{\frac{E_{k}}{2H_{\mathrm{ab}}}\sum_{i=1}^{m}P_{d_{i}}}{\sqrt{1+{\left(\frac{E_{k}}{2H_{\mathrm{ab}}}\right)}^{2}{\left(\sum_{i=1}^{m}P_{d_{i}}\right)}^{2}}}$$

We refer to Equaiton 21 as the two-state model (TSM) for mQCA gates along this paper. The *E*_*k*_ and the *H*_*ab*_ are the only parameters of the TSM. Once the geometrical parameter *l* is determined, experimentally or from theoretical calculations, the Kink energy can be calculated using Equation 15. The electronic coupling matrix element, *H*_*ab*_, can be calculated using various quantum chemistry techniques [34,38-41] or obtained via spectroscopic experiments, including absorption [42,43], EPR [44], and ultraviolet photoelectron spectroscopy [45]. As we will present, the parameter *μ = E*_*k*_*/2H*_*ab*_ plays an important role in the accuracy of the TSM. It is also the slope of the switching response function at the origin i.e.,

(22) $$\frac{\partial P_{o}}{\partial \sum_{i=1}^{m}P_{d_{i}}}{|}_{\sum_{i=1}^{m}P_{d_{i}}=0}=\mu$$

## Results and discussion

The chemistry of mixed-valence complexes has received considerable attention recently in mQCA device implementation, where the intramolecular electron transfer and charge localization at redox sites are the important key factors. Mixed-valence compounds contain more than one redox site in the same molecule or molecular unit. Simple model molecules for two-dot cells are the *π-σ-π* mixed-valence types, which were proposed by Aviram and studied later by Hush [3,46]. In the Aviram's model molecule (1, 4-diallyl butane cation), the two π-allyl groups form two redox centers and are connected by a σ-butyl bridge. One of the allyl groups is a neutral radical, while the other one is anionic (or cationic). The possibility of charge localization in some mixed-valence mQCA candidate molecules has been examined theoretically as well as experimentally [15,47,48]. Advances in quantum chemistry in the past half century provide reliable methods to explore the electronic structure of molecules; however, many of the *ab initio* techniques fail in describing charge distribution in mixed-valence complexes. The unrestricted Hartree-Fock method overestimates the charge localization due to the neglect of electron correlation effects [49]. In the density functional theory (DFT) method, the exchange potential defined in hybrid functional leads to underestimation of charge localization [47,49]. The complete active space self-consistent field (CASSCF) method [50,51] is believed to be the most reliable for describing charge distribution in mixed-valence complexes [50]. However, the multi-determinant CASSCF calculations scale with the system size, which makes this method highly demanding in computer time and memory. The number of Slater determinants has factorial dependence on both the number of active electrons and particularly on the number of active orbitals generating many-electron configurations (full configuration interaction (CI) within the active space). This is much more significant than any dependence on the number of one-electron basis functions. The number of Slater determinants in a full CI calculation is given by:

(23) $$n_{\mathrm{Slater}}=\left(\begin{array}{l} M\\ N_{\alpha } \end{array}\right)\left(\begin{array}{l} M\\ N_{\beta } \end{array}\right)$$

where *M* is the number of active orbitals, *N*_*α*_ and *N*_*β*_ are the numbers of active electrons with α- and β-spins, respectively, and the quantities in parentheses are binomial coefficients:

(24) $$\left(\begin{array}{l} a\\ b \end{array}\right)=\frac{a!}{b!(a-b)!}$$

**Figure 4:**
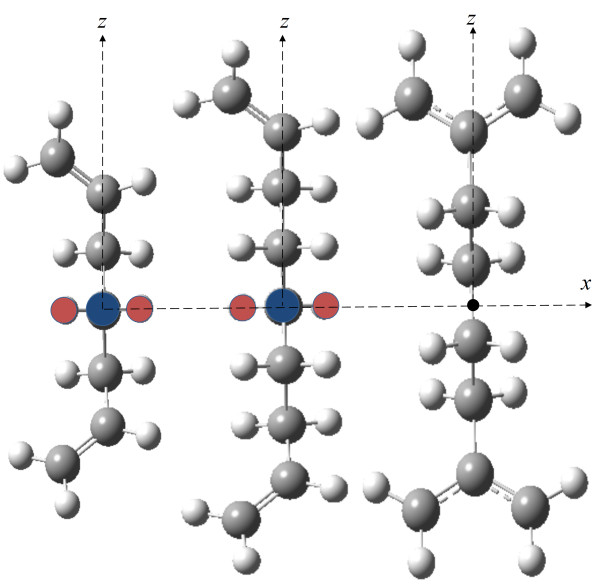
**Geometry of the molecules we used in our calculations.** 1, 6-heptadiene, 1, 8-nonadiene, and 1, 4-diallyl butane radical monocations from left to right. In the first two molecules, coordinates of the three highlighted atoms have been set to *xy* plane, while the coordinates of the central carbon have been set to origin. For 1, 4-diallyl butane, the origin has been set in the middle of the bond between the two central carbon atoms.

We present full quantum chemistry calculations of the steady-state output polarization of the universal MinV model gate serving as INV and three-input MinV gates. The results based on full quantum chemical calculations are compared to the results obtained from the TSM. The π-σ-π mixed-valence type molecules, descended from Aviram's original idea are analyzed. These molecules include 1, 6-heptadiene, 1, 8-nonadiene, and 1, 4-diallyl butane radical cations, which will be referred to as molecule 1, molecule 2, and molecule 3 in this paper, respectively (Figure 4). We optimized the geometry of these monocations using the DFT/B3LYP method. The dot-dot distance, *l*, in these molecules is between 0.5 to 0.8 Å. Bistability and electron localizability of these molecules have been studied in [3,46,49].

Koopmans' theorem [52] has found extensive application in calculation of the ET matrix element, *H*_*ab*_ for symmetric molecules. Under the two-state approximation, *H*_*ab*_ is related to adiabatic energies of the ground and first excited state (*E*_*1*_ and *E*_*2*_) as [34]

(25) $$H_{\mathrm{ab}}=(1/2)(E_{2}^{}-E_{1}^{})\sin2\eta$$

When no driver is present or the sum of the input drivers is zero, *P*_*o*_ = 0; and from Equation 18 it is clear that *Cos2η* = 0, thus

(26) $$H_{\mathrm{ab}}=(1/2)(E_{2}-E_{1})$$

According to the one electron Koopmans' theorem, the ionization potential of the highest occupied molecular orbital (HOMO) and HOMO-1 can be expressed in terms of the molecular orbital (MO) energies, i.e., [38-41]

(27) $$I_{\mathrm{HOMO}}=-\epsilon_{\mathrm{HOMO}}$$

(28) $$I_{HOMO-1}=-\epsilon_{HOMO-1}$$

Inserting Equations 27 and 28 into Equation 26, the electronic coupling element is obtained in terms of the MO energies as [38-41]

(29) $$H_{\mathrm{ab}}=(1/2)(\epsilon_{\mathrm{HOMO}}-\epsilon_{HOMO-1})$$

The state-averaged CASSCF (SA/CASSCF) method [53] can be used to calculate the electronic coupling element of asymmetric molecules. One can obtain *H*_*ab*_ by calculating the energies of the ground and first excited states and use them in Equation 26 within the two-state approximation. We have calculated the electronic coupling elements using both methods. The calculations for molecule 1 and 2 were based on SA/CASSCF(34), and the calculations for molecule 3 were based on SA/CASSCF(56). In 1, 4-diallyl butane cation, the allyl π-bonds are aromatic, and the active space is extended to five electrons in six orbitals. The calculated ET matrix elements have been compiled in Table 1. The Kink energies of the molecules have also been calculated using Equation 15. Table 1 includes all required parameters of the TSM. All calculations reported here were performed in Gaussian 09 [54], using 6-31 G(d) basis set. Various basis sets have been extensively tested to examine the basis set dependency of the results. Application of larger basis sets did not significantly influence the energy difference between the ground state and the first excited state. The results from the two methods are in good agreement. The ET matrix element, *H*_*ab*_ decays exponentially with dot-dot distance, *l*[55]. The dot-dot distance for molecule 3 is less than that of molecule 2; however, the *H*_*ab*_ has been remarkably decreased. This is due to the symmetry of this molecule and the aromatic bonds of the radical allyls. The geometrical parameter, *l*, and the type of head groups, play an important role in determining the ET matrix element. To obtain a more accurate electronic coupling element, the overlap integral, *S*_*ab*_, should be taken into account as described in the work of Farazdel et al [56]. Aviram [3] has obtained a negligible overlap integral for molecule 3 with dot-dot distance of 7 Å. In mQCA, the electron transfer drama should have a little effect on the geometric parameters [4]. Consequently, candidate molecules should possess fast electron transfer reactions, and the relaxation of nuclear degrees of freedom can be ignored. Table 1 also lists the changes in the head groups' π-bonds (Δζ) as a consequence of ET reactions. It is seen that ET reactions in molecule 3 should be faster compared to the other two molecules.

**Table 1:** Two-state model parameters for the used molecules

**Molecule (cation)**	^**1**^**H**_**ab**_**(eV)**	^**2**^**H**_**ab**_**(eV)**	***l*(nm)**	**E**_**k**_**(eV*)***	**|μ|**	^***3***^***Δζ*(Å)**
1,6-heptadiene	0.310	0.368	0.56	−0.7531	1.023	0.06969
1,8-nonadiene	0.14	0.12	0.83	−0.5081	2.117	0.07002
1,4-diallyl butane	0.00707	0.00693	0.7	−0.6025	43.04	0.00905

^*1*^*H*_*ab*_ has been calculated based on the Koopmans' theorem and DFT/B3LYP method.

^*2*^*H*_*ab*_ has been calculated based on the SA/CASSCF method.

^3^Calculations have been done based on CASSCF method.

### **INV gates**

The INV gate in two-dot mQCA is the nucleus of all other gates. Once its operation and switching properties are clearly understood, the properties of more intricate structures such as multidriver MinV gates can be derived from extrapolating the results obtained from the inverters, based on the additivity relation (Equation 20). The analysis of inverters can be extended to explain the behavior of more complex gates, which in turn form the building blocks for modules such as adders, multipliers, and processors. Table 2 compares the output polarizations of the INV gates, obtained from full quantum chemistry calculations and the TSM. The normalized dipole moments (Equation 2) of the monocations adjacent to fixed inputs (point charges as fixed drivers) have been calculated based on SA/CASSCF(3,4) for molecule 1 and 2, and SA/CASSCF(5,6) for molecule 3. The root mean square errors (RMSE) of the results obtained from the two methods have been calculated. The RMSE decreases with the increase of the *μ* parameter (or decrease of the ET matrix element, *H*_*ab*_), determining the degree of agreement between the results. The saturation polarization of the output is also dependant on the *μ* parameter. For INV gates, it is obtained by setting the sum of the input drivers to one in Equation 21 as

(30) $$\left|P_{o}{}^{\mathrm{sat}}\right|=\frac{1}{\sqrt{1+1/\mu^{2}}}$$

**Table 2:** INV gates

	**1,6-heptadiene**		**1,8-nonadiene**		**1,4-diallyl butane**	
***P***_***d***_	***P***_***o***_	***P***_o_^*^	***P***_***o***_	***P***_o_^*^	***P***_***o***_	***P***_o_^*^
0.0	−0.068	0	−0.058	0	−2.3 E-05	0
0.1	−0.126	−0.101	−0.217	−0.207	−0.987	−0.974
0.2	−0.191	−0.200	−0.398	−0.389	−0.987	−0.993
0.3	−0.260	−0.293	−0.534	−0.536	−0.989	−0.997
0.4	−0.323	−0.378	−0.630	−0.646	−0.990	−0.998
0.5	−0.378	−0.455	−0.695	−0.726	−0.992	−0.998
0.6	−0.423	−0.523	−0.740	−0.785	−0.994	−0.999
0.7	−0.460	−0.582	−0.771	−0.828	−0.996	−0.999
0.8	−0.490	−0.633	−0.793	−0.861	−0.997	−0.999
0.9	−0.513	−0.677	−0.808	−0.885	−0.998	−0.999
1.0	−0.531	−0.715	−0.818	−0.904	−0.999	−0.999
RMSE^*^	0.104		0.050		0.006	

When there is a single driver, the change in the sign of the input will result in a change in the sign of output polarization. The negative inputs have been omitted to reduce the size of the table.

*P*_o_ and *P*_o_^*^ denote the calculated output polarizations based on the SA/CASSCF and TSM methods respectively.

RMSE^*^ represents the root mean square errors of *P*_*o*_ and *P*_o_^*^.

Equation 30 shows that the saturation polarization of the output increases with the increase of *μ*. This is also evident from the results in Table 2.

### **Two-driver devices**

In the model MinV gate, the number of input drivers (*m*) should be odd. No logic operation is performed when *m* is even. However, neglecting the logic, the two-driver device is an appropriate small model system for studying the additivity relation, and how the accuracy of the TSM is influenced by the number of drivers. Here, the MinV gate is probed when only the two input drivers *d*_*1*_ and *d*_*3*_ are present (Figure 3a). We have calculated the normalized dipole moments of the gates' outputs based on the SA/CASSCF calculations. The output polarizations have also been calculated by the TSM. The results obtained from the two methods are compiled in Table 3, which are in good agreement. The conclusions from analysis of the INV gates can be extrapolated to two-driver devices as well. Compared to INV gates, the increase in the RMSE of the two-driver devices, composed of molecule 1 or 2, is mainly attributed to the asymmetric head groups. In other words, the effect of d_1_ on the head groups is different from that of d_3_, where *P*_*d1*_*= P*_*d3*_. In molecule 3, the allyl head groups are symmetric, and the TSM error mainly arises from the classical approximation of the intercellular interactions. It is important to note that the output polarization of the two-driver devices can be calculated by employing the additivity relation on the output polarizations of the INV gates. The additivity relation has been validated for the SA/CASSCF method as well. Through full quantum chemistry calculations, the output polarizations of the two-driver devices were obtained. We also used the results of the INV gates, *P(P*_*d1*_*+ P*_*d2*_*)* from Table 2, to examine the additivity relation for SA/CASSCF method. As expected, RMSE is highly dependent on the symmetry of the head groups. Unlike molecule 1 and 2, for the case of molecule 3, each driver has an exactly same effect on the head allyl groups, which leads to smaller RMSE. We also highlight that employing additivity relation decreases the computational cost of SA/CASSCF calculations. Table 2 and Table 3 also show how the accuracy of the TSM is affected by the number of input drivers. It is seen that RMSE is approximately doubled when the number of input drivers is scaled up by a factor of two.

**Table 3:** Two-driver devices

***P***_**d1**_	***P***_**d2**_	**1,6-heptadiene**		**1,8-nonadiene**		**1,4-diallyl butane**	
		***P***_**o**_**(*P***_**d1**_**,*P***_**d2**_**)**	***P***_**o**_**(*P***_**d1**_**+*P***_**d2**_**)**	***P***_**o**_**(*P***_**d1**_**,*P***_**d2**_**)**	***P***_**o**_**(*P***_**d1**_**+*P***_**d2**_**)**	***P***_**o**_**(*P***_**d1**_**,*P***_**d2**_**)**	***P***_**o**_**(*P***_**d1**_**+*P***_**d2**_**)**
0	0	−0.068	−0.068	−0.058	−0.058	−0.001	−0.001
0.2	0.2	−0.316	−0.323	−0.625	−0.630	−0.998	−0.992
0.4	0.2	−0.431	−0.423	−0.753	−0.740	−0.994	−0.987
0.6	−0.2	−0.326	−0.323	−0.651	−0.630	−0.998	−0.992
0.8	0.2	−0.560	−0.531	−0.840	−0.768	−0.985	−0.979
1	−0.4	−0.383	−0.423	−0.718	−0.740	−0.995	−0.988
0.4	−0.2	−0.204	−0.191	−0.454	−0.398	−0.999	−0.993
1	−0.2	−0.469	−0.490	−0.788	−0.793	−0.990	−0.984
0.6	−0.8	0.090	0.191	0.075	0.398	0.997	0.991
RMSE		0.038		0.112		0.005	
RMSE^*^		0.137		0.108		0.014	

*P*_o_(*P*_d1_, *P*_d2_) and *P*_o_(*P*_d1_ + *P*_d2_) denote the calculated output polarizations based on the SA/CASSCF method.

RMSE has been calculated based on *P*_o_(*P*_d1_,*P*_d2_) and *P*_o_(*P*_d1_ + *P*_d2_).

RMSE^*^ has been calculated based on *P*_o_(*P*_d1_, *P*_d2_) and *P*_o_^*^(*P*_d1_ + *P*_d2_) from Table 2.

### **Three-input MinV gates**

The multidriver MinV gate is a universal gate in the two-dot mQCA. The output polarizations of these gates with three fixed input drivers are shown in Table 4. This table also shows that the output polarizations of the MinV gates can be obtained from extrapolating the INV output polarizations using the additivity relation. For the model molecules, considering the spatial location of the d_2,_ the effect of d_2_ on the head groups is different from the same effect of d_1_ and d_3_, while in the TSM, drivers with equal polarizations have same effects on the head groups and are treated the same. Quantum chemical calculations show that despite the equal sum of the input polarizations, the output polarizations are not equal particularly when the sum of the drivers is zero. Table 4 also displays that the SA/CASSCF method returns different output polarizations, while the sum of the input polarizations is zero. This is the main reason of the decrease in the accuracy of the results obtained from the TSM for MinV gates. Ignoring these points by avoiding the null state logic occurrence, the two-state approximation results are fairly in good agreement with the quantum chemical calculations. Table 2 and Table 4 show how the accuracy of the two-state model is decreased with the number of drivers. It is seen that RMSE is tripled when the number of input drivers is scaled up by a factor of three.

**Table 4:** **Three-driver*MinV*gates**

***P***_***d1***_	***P***_***d2***_	***P***_***d3***_	**1,6-heptadiene**		**1,8-nonadiene**		**1,4-diallyl butane**	
			***P***_***o***_	***P***_o_^*^	***P***_***o***_	***P***_o_^*^	***P***_***o***_	***P***_o_^*^
0	0	0	−0.005	0	−0.015	0	−0.001	0
0.2	0.2	1	−0.605	−0.819	−0.826	−0.947	−0.993	−0.999
0.4	0.2	0.6	−0.596	−0.775	−0.825	−0.930	−0.996	−0.999
0.6	−0.2	1	−0.610	−0.819	−0.826	−0.947	−0.993	−0.999
0.8	0.2	−1	−0.064	0	−0.289	0	−0.992	0
1	−0.4	1	−0.617	−0.853	−0.828	−0.959	−0.989	−0.999
0.4	−0.2	−0.2	−0.061	0	−0.143	0	−0.954	0
1	−0.2	1	−0.627	−0.878	−0.832	−0.967	−0.984	−0.999
0.6	−0.8	−0.2	0.197	0.378	0.377	0.646	0.988	0.998
−0.4	−0.8	−0.8	0.639	0.898	0.837	0.973	0.993	0.999
0.6	0.8	1	−0.617	−0.950	−0.836	−0.981	−0.969	−0.999
1	1	1	−0.634	−0.926	−0.830	−0.987	−0.957	−0.999
RMSE^*^			0.213		0.162		0.397	
RMSE^**^			0.244		0.153		0.019	

*P*_o_ and *P*_o_^*^ denote the calculated output polarizations based on the SA/CASSCF and TSM methods, respectively.

RMSE^*^ has been calculated based on *P*_o_(*P*_d1_, *P*_d2_, *P*_d3_) and *P*_o_^*^(*P*_d1_ + *P*_d2_ + *P*_d3_).

RMSE^**^ has been recalculated when points with *P*_o_^*^= 0 have been omitted.

## Conclusions

Molecular QCA gates are the building blocks of more complex modules. Probing molecular devices requires quantum chemical calculations, which are challenging as the molecular system grows in size. A semi-classical model was derived directly from the two-state approximation in the ET theory, serving as a device for studying mQCA gates. This model is very similar to the two-state model which is currently the core of the QCADesigner simulation engine for solving circuits based on metallic *QCA*. The range of applications and limitations of this model for mQCA gates was investigated carefully. The parametric TSM can be used to study more complex mQCA gates composed of practical candidate mixed-valence molecules, where exploiting the SA/CASSCF method is of high computational cost. A complete set of logic gates were implemented within the two-dot mQCA framework. These gates include INV and MinV gates, which provide a small molecular test bench, making further analysis by quantum chemistry methods, particularly SA/CASSCF, practical. The INV gate was studied as a nucleus of all other gates. It was also presented that output polarizations of all other gates can be derived from extrapolating the results obtained from inverters based on the additivity relation. We compared the results obtained from the TSM to those obtained from SA/CASSCF calculations for INV and MinV gates. The degree of agreement between the TSM and quantum chemical calculations is highly dependent on the *μ* parameter and the symmetry of the head groups. Additionally, application of the additivity relation for CASSCF method can in turn reduce the computational cost. It is important to note that we did not address questions of surface attachment, input/output, clocked control, layout, and patterning, which are the requirements of a practical QCA system. Moreover, we did not consider the relaxation of nuclear degrees of freedom associated with electron transfer. It is presented that for mQCA, the electron localization and Coulombic interactions play the key roles, and nuclear positions can be considered frozen (nuclear relaxation even assists charge localization) [4]. Although we limited our focus on the two-dot mQCA, it merits highlighting that the model can also be used for four-dot cells, since they can be considered as double two-dot cells. Our focus was on the mQCA gates as building blocks of circuits. The two-state model may be applied to simulate mQCA circuits as well, as it is currently used iteratively for simulation of metallic QCA circuits in the QCADesigner. However, to determine the additive error resulting from exploiting the two-state model for solving mQCA circuits, further quantum chemical calculations on the mQCA clocked circuits composed of several molecules are required, which are extremely challenging at the time, and have not been addressed in this paper.

## Competing interests

The authors declare that they have no competing interests.

## Authors' contributions

ER developed the theories and carried out the quantum chemical calculations. SMN supervised the project. All authors read and approved the final manuscript.
